# Antimicrobial Resistance of Bacterial Agents of the Upper Respiratory Tract of School Children in Buea, Cameroon

**DOI:** 10.3329/jhpn.v26i4.1881

**Published:** 2008-12

**Authors:** R.N. Ndip, E.A. Ntiege, L.M. Ndip, G. Nkwelang, J-F. T.K. Akoachere, Akenji T Nkuo

**Affiliations:** 1 Department of Biochemistry and Microbiology, Faculty of Science, University of Buea, Box 63, Buea, Cameroon; 2 Department of Biochemistry and Microbiology, Faculty of Science and Agriculture, University of Fort Hare, P/Bag X 1314, Alice 5700, South Africa

**Keywords:** Antibiotic resistance, Antibiotics, Bacteria, Child, Drug resistance, Microbial, Influenza, Microbial susceptibility tests, Pneumonia, Respiratory tract infections, Cameroon

## Abstract

The study was aimed at determining bacterial agents of the upper respiratory tract and the susceptibility patterns of isolates to antibiotics. In total, 200 throat swabs were obtained from students attending different boarding schools within the Buea Municipality and screened to obtain the prevalence of respiratory pathogens and to understand the antibiotic susceptibility patterns of isolates using standard microbiological procedure and the disc-diffusion test. Of the 200 samples screened, 112 (56%) had positive cultures with the dominant bacterial pathogens being *Haemophilus influenzae* (20%), followed by *Streptococcus pneumoniae* (15%), *Klebsiella pneumoniae* (11%), and *Staphylococcus aureus* (10%). Although 56% of the isolates were recovered from females compared to 44% from males, the difference was not statistically significant (p>0.05). Sixty-seven percent of the pathogens were isolated from the age-group of 10-13 years, 19.6% from the age-group of 14-17 years, and 12.5% from the age-group of 18-21 years. Antibiotic susceptibility testing revealed that gentamicin (92%) and cefuroxime (88.4%) were the most effective antibiotics against the isolates. Generally, susceptibility ranged from 0% to 92% depending on the antibiotic and the species of microorganism. Penicillin had the highest (100%) resistance to all the isolates. The findings revealed that students living in boarding schools in the Buea Municipality were at risk of acquiring upper respiratory tract infections from their peers since the upper respiratory tract of more than 50% of the students was colonized with respiratory pathogens. Although multidrug-resistant strains of organisms were identified, gentamicin and cefuroxime are recommended as the first-line antibiotics of choice against the pathogens. There is, therefore, a need for surveillance of nasopharyngeal carriage of resistant strains of these organisms, especially *H. influenzae* in unhealthy school children since the vaccine is yet to be introduced in Cameroon. The findings have clinical and epidemiological significance.

## INTRODUCTION

Respiratory tract infections (RTIs), which involve the upper or lower respiratory tract, frequently occurs after birth (1). RTIs, such as sore throat, earache, laryngitis, common cold, otitis media, sinusitis, and mastoiditis, are the most frequently-occurred infections of all human diseases and have been frequently documented (1,2). Since these infections often seem trivial, they are more commonly discounted as being mere temporary inconveniences that cause transient discomfort (3). Recurrent RTIs (RRTIs) in children constitute a serious problem worldwide. Some children experience considerable morbidity as a result of RRTIs and receive repeated courses of antibacterials that are not effective against viral infectious agents and can increase bacterial resistance. Furthermore, the direct and indirect costs of RRTIs to the community are substantial. In most tropical countries, acute RTIs stand as a leading cause of hospitalization and death (2).

There is a widespread agreement that viruses are the principal initiators of RTIs (4). It has also been reported that pneumonia due to secondary bacterial infections is the most important complication of the lower respiratory tract and that surveillance of acute RTIs in defined populations to monitor prevailing pathogens and to determine population groups at special risk are important for taking preventive measures (5,6). The main bacterial pathogens recovered from complicated influenza virus infections include *Haemophilus influenzae, Staphylococcus aureus, Streptococcus pneumoniae, Klebsiella pneumoniae*, and *Streptococcus pyogenes* (4,7). *Pseudomonas aeruginosa* and some members of the Enterobacteriaceae may also be implicated (8,9).

Respiratory diseases have overtaken diarrhoea as the most frequent cause of death among children in developing countries, with *S. pneumoniae* among others being one of the main pathogenic species (10). This author has earlier reported that pneumonia in particular is now the leading cause of childhood death, taking the place of diarrhoeal diseases and is also responsible for at least 40% of cases, where children are taken to clinics or hospitals. *S. pneumoniae* carriage has been reported to vary from 9% to 72% in different studies in sub-Saharan Africa (11-14).

*H. influenzae* type b (Hib) has been reported to account for 20% of cases of radiologically-confirmed pneumonia in children in sub-Saharan Africa (15,16). In Kenya, for example, invasive H. influ- *enzae*-associated disease is responsible for 5% of inpatient deaths among children (15). RTIs in dormitory institutions have long been thought to be a dominant health problem because infected students may transmit causative pathogens to other students and even teachers, since they stay together for quite long periods of time, coupled with the poor ventilation system, which is one of the characteristics of the developing world (17).

Cases of acute RTIs are known to respond to antibiotics. Severe pneumonia and meningitis have been reported to respond to chloramphenicol and benzylpenicillin, and mild pneumonia to trimethoprim-sulphamethoxazole, ampicillin, or amoxicillin (15). However, the overuse and misuse of antibiotics for upper RTIs in children is widespread and fuelled by public attitudes and expectations heralding the emergence of resistance (18). Resistance levels of up to 84% have been reported for co-trimoxazole, 52% for penicillin, and 25% for ampicillin (12,19). It has also been widely acclaimed that the susceptibility of pathogens to antibiotics varies with time and geographical location (20,21). The ever-increasing tendency to purchase drugs over-the-counter in our environment heralds the emergence of resistant strains of pathogens posing a great problem in the treatment and control of such pathogens (22). It is, therefore, necessary to study the susceptibility patterns of these isolates to some commonly-used and relatively-reserved antibiotics in Cameroon to update knowledge on the use of these antimicrobial agents in the management of RTIs.

In the literature, the incidence of RTIs, such as pneumonia and meningitis, has often been estimated in relation to vaccine studies, which unfortunately have never been conducted for these pathogens in Cameroon. There are, therefore, no estimates of incidence, mortality, or hospital burden for the pathogens. Thus, the impact of childhood bacteraemia is largely unknown in Cameroon. In Buea, there are no reports on the spectrum of bacteria incriminated in RTIs in boarding schools and their antibiogram, although students in these institutions frequently come down with such related symptoms and ailments. It is against this background that the bacterial agents of RTIs and their antibiogram were determined in an attempt to provide baseline data for clinical management and epidemiological surveys.

## MATERIALS AND METHODS

### Study design and subjects

The study was carried out in Buea, the south-west province of Cameroon. Buea is about 1,000 metres above sea level and has an average yearly temperature of 15 oC with relatively high humidity, which are predisposing factors for RTIs (23).

The study was undertaken in three highly-populated schools in the Buea Municipality. The schools were chosen because they had boarding facilities that allow students to have more intimate contact than day schools. Female dormitories were, on average, more congested than male ones. The ratio of females to males in the dormitories was 3:1. The female dormitories, on average, measured about 15×7 metres with eight windows. With an original capacity of 40 students per dormitory, about 60 students actually resided in each dormitory at the time of the study. The male dormitories, on average, had dimensions of 12×6 metres with 10 windows. Although the original capacity was for 25 students, 35 students resided there at the time of the study.

In total, 200 students took part in the study, of which 93 were males and 107 were females. The study subjects were aged 10-21 years with a mean age of 15 years. All the subjects were informed of the aim of the study through their school authorities and were assured that all information supplied by them will remain confidential. Their verbal consent and cooperation to participate in the study was then solicited. The school authorities preferred not to reveal the true identity of their respective schools, but identified them by letters corresponding to the three schools, respectively, A, B, and C. The distribution of the students per school was 65, 69, and 66 for school A, B, and C respectively.

Ethical approval was obtained from the South West Provincial Delegation of Public Health.

The criteria for inclusion in the study were: the student must be suffering from RTIs, referred to clinics or hospitals, and must not have been taking antibiotics of any kind at least for the two weeks preceding the study. The students presented RTIs, including sore throat, otitis media, runny nose, sinusitis, cough, and pneumonia. The large majority (78%) of them had runny nose and sore throat while pneumonia was the least noted. The symptoms lasted for 3-14 days. The study continued from October 2004 to July 2005.

### Bacterial isolates

Trained personnel of the infirmary of the schools collected 200 throat swabs. The specimens were transported to the Microbiology Laboratory of the Department of Life Sciences, University of Buea, for analysis. The specimens were collected in seven months from November 2004 to May 2005 spanning both dry and early wet seasons.

A loopful of each sample was inoculated onto blood agar, chocolate agar, and eosin methylene blue agar. The inoculated plates were incubated at 37 oC for 24-48 hours aerobically, except for chocolate agar, in which the plates were incubated for 24-48 hours at 37 oC in an atmosphere of 5-10% CO2 (24). After incubation, macroscopic and microscopic examinations of colonies on plates were carried out, and suspect colonies were subcultured on appropriate solid culture media for purification. They were later subcultured on appropriate slants and stored at 4 oC for further analysis.

Pure cultures were presumptively identified based on their cultural and morphological characteristics on selective and differential media (24). Standard microbiological techniques and biochemical tests using the API 20E kit (Biomerieux, SA, France) were employed to confirm isolates following the instructions of the manufacturer.

### Antibiotic susceptibility testing

The Kirby-Bauer disc-diffusion test was used as previously described (25). Briefly, a small inoculum of each bacterial isolate was emulsified in three-mL sterile normal saline in Bijou bottles, and the density was compared with a barium chloride standard (0.5 McFarland). A sterile cotton swab was dipped into the standardized solution of bacterial cultures and used for evenly inoculating Mueller-Hinton plates (Biotec, England) and allowed to dry. Thereafter, antibiotic discs with the following drug contents—ampicillin (10 μg), chloramphenicol (10 μg), cefuroxime (30 μg), cefazolin (30 μg), co-trimoxazole (25 μg), erythromycin (10 μg), gentamicin (10 μg), and penicillin (10 IU) (Oxoid, England)—were placed on the plates, spacing them well to prevent the overlapping of inhibition zones. These antibio-tics were selected based on prescription practices for RTIs in our locality and from the literature (26). The plates were incubated at 37 oC for 24 hours, and the diameters were compared with recorded diameters of the control organism—*Escherichia coli* ATCC 25922—to determine susceptibility or resistance (22).

### Statistical analysis

Data obtained were analyzed using the Epi Info software (version 5) (Centers for Disease Control and Prevention, USA). Categorical data (comparison of schools, sex, and age-groups) were compared by chi-square. P values of <0.05 were considered to be statistically significant.

## RESULTS

The prevalence of bacteria isolated from throat swabs of students attending some schools in Buea, Cameroon, is presented in Table 1. Fifty-six percent of the samples analyzed had positive cultures. Four main species of bacteria were identified: *H. influenzae* (20%), *S. pneumoniae* (15%), *K. pneumoniae* (11%), and *S. aureus* (10%). The organisms were isolated more (62.12%) from students in school C (p>0.05) than from those in the other two schools.

**Table 1.** Prevalence of pathogens in the different schools

Species	School*	Isolates	
		No.	%
*H. influenzae*	A	9 13.8
	B	17 24.4
	C	14 21.2
*K. pneumoniae*	A	6 9.4
	B	8 11.6
	C	8 12.1
*S. aureus*	A	8 12.3
	B	5 11.6
	C	7 10.6
*S. pneumoniae*	A	10 15.4
	B	8 11.6
	C	12 18.2
Total		112 56

*The number of students examined from the respective school: A=65, B=69, and C=66

Of the 112 isolates identified, 56% were recovered from females compared to 44% from males. *H. influenzae* recorded the highest prevalence in both males (16.1%) and females (23.3%) while *S. aureus* occurred least with the prevalence of 9.6% and 10.2% among males and females respectively. Table 2 shows the prevalence of the isolates in the different age-groups. Of the 112 positive cultures recorded, the percentage of isolation in the various age-groups was 67.9% for the age-group of 10-13 years, 19.6% for the age-group of 14-17 years, and 12.5% for the age-group of 18-21 years (p<0.05).

**Table 2.** Prevalence of isolates by age (years)

Species	Age-groups (years)*						Total	
	10-13		14-17		18-21			
	No.	%	No.	%	No.	%	No.	%
*H. influenzae*	40	32.8	0	0	0	0	40	20
*K. pneumoniae*	15	12.3	6	9.4	1	7.1	22	11
*S. aureus*	10	8.2	7	11	3	21.4	20	10
*S. pneumoniae*	11	9.0	9	7.4	10	71.4	30	16
Total	76	67.9	22	19.6	14	12.5	112	56

*The number of subjects in each age-category: 10-13 years=122; 14-17 years=64; 18-21 years=14

The susceptibility patterns of isolates to various antibiotics are shown in Table 3. The susceptibility ranged from 0% to 92% depending on the antibiotic and species of microorganism. The lowest susceptibility (0%) was observed for all the isolates to penicillin and *H. influenzae* to co-trimoxazole (23.2%) respectively. Gentamicin (92%) and cefuroxime (88.4%) generally exhibited good activity against the isolates. All the isolates were resistant to at least one antibiotic. The overall resistance rates were generally low for gentamicin (8%), cefuroxime (11.6%), and cefazolin (22.3%) respectively. However, penicillin had the highest resistance (100%).

**Table 3.** Antibiotic susceptibility patterns of isolates

Isolates and number tested	No. of isolates sensitive and resistance to															
	AMP		C		E		CXM		COT		CZ		GN		P	
	R	S	R	S	R	S	R	S	R	S	R	S	R	S	R	S
*H. influenzae*	(n=40)	24	16	12	28	12	28	1	39	40	0	6	34	2	38	40	0
(60)	(40)	(30)	(70)	(30)	(70)	(2)	(98)	(100)	(00)	(15)	(85)	(4)	(96)	(100)	(00)
*K. pneumoniae*	(n=22)	13	9	9	13	6	16	4	18	13	9	6	16	1	21	22	0
(60)	(40)	(40)	(60)	(25)	(75)	(20)	(80)	(60)	(40)	(25)	(75)	(2)	(98)	(100)	(00)
*S. aureus*	(n=20)	14	6	6	14	5	15	3	17	12	8	4	16	1	19	20	0
(70)	(30)	(30)	(70)	(28)	(72)	(15)	(85)	(60)	(40)	(25)	(75)	(2)	(98)	(100)	(00)
*S. pneumoniae*	(n=30)	18	12	14	16	9	21	5	25	21	9	9	21	5	25	30	0
(60)	(40)	(45)	(55)	(30)	(70)	(15)	(85)	(70)	(30)	(30)	(70)	(15)	(85)	(100)	(00)
Total	69	43	41	71	32	80	13	99	86	26	25	87	9	103	112	0
	(62)	(38)	(36.7)	(63.3)	(28.6)	(71.4)	(11.6)	(88.4)	(76.8)	(23.2)	(22.3)	(77.7)	(8)	(92)	(100)	(0)

Figures in parentheses indicate percentages; AMP=Ampicillin (10 μg); C=Chloramphenicol (10 μg); COT=Co-trimoxazole (25 μg); CXM=Cefuroxime (30 μg); CZ=Cefazolin (30 μg); GN=Gentamicin (10 μg); E=Erythromycin (10 μg); P=Penicillin (10 IU); R=Resistance; S=Sensitive

## DISCUSSION

The study focused on the prevalence and antibiogram of bacteria causing upper RTIs among students in some schools in the Buea Municipality. *H. influenzae* was the most frequently-isolated pathogen (20%). The isolation rates of 15%, 11%, and 10% were also noted for *S. pneumoniae, K. pneumoniae*, and *S. aureus* respectively. Previous studies had incriminated these organisms as significant causes of RTIs (20,27-29). Our results are, therefore, in harmony with the findings of these studies. This may, therefore, suggest that the students in boarding schools in the Buea municipality are at risk of acquiring RTIs from symptomatic individuals via the sharing of common refectory and dormitory utensils

The isolation of these pathogens from the throat of students with RTIs do not only place their peers at risk but also their teachers with whom they interact in class and other areas within the school. The high prevalence of isolates could be due to the fact that many students are malnourished, debilitated, and young and also coupled with the fact that the cold climate of Buea (average temperature at the time of study was 15 oC) is favourable to the initiators (viruses) of RTIs as previously suggested (20). We also speculate that the teachers may constitute a source of contamination for the students. Although we did not screen them, we had previously demonstrated a high prevalence of these pathogens in subjects in the neibourhood of Buea where most of these teachers reside (23).

In a study in Saudi Arabia, *H. influenzae* was the predominant organism isolated from patients with lower RTI (27). In another study, El-Sheikh showed that *H. influenzae* was the most frequently-incriminated bacterial pathogen among pilgrims to the Haj (20). This is further supported by another study in Japan (29). Our results are, therefore, in harmony with findings of these studies. Although we did not type *H. influenzae*, the prevalence of *H. influenzae* carriage among school children suggests that the transmission of these organisms from children of this age to young unvaccinated children is likely to continue for several years since Hib vaccine has not been introduced in Cameroon. This is supported by findings of the study of Cowgill *et al*. in rural Kenya, in which the introduction of Hib vaccine did not result in rapid elimination of Hib disease (30). Noteworthy in the present study is the finding that *S. pyogenes,* which is known to be a leading cause of RTI (17), was not isolated. In a similar study in Buea however, we reported a prevalence (20.1%) of *S. pyogenes* (23), and in Yaoundé, Cameroon, Koulla-Shiro *et al*. reported the prevalence of *S. pyogenes* in patients with community-acquired pneumonia (28). According to Spencer and Philip, negative cultures may be attributed to other aetiologic agents or patients already on chemotherapy (31). The frequent use of antibiotics, which may be self-administered by patients or bought over-the-counter from pharmacies, may result in failure to isolate any potential pathogen, despite strong clinical evidence of chest infection (32). We may not be able to categorically ascertain the non-isolation of *S. pyogenes,* in the present study, but speculate that one reason may be because antibiotics are indiscriminately taken in our environment (22), although our inclusion criteria excluded students who were on antibiotics two weeks prior to sampling.

There were no significant differences in the prevalence of the pathogens in the various schools and with respect to the sexes (p>0.05), suggesting that RTI is not school- or sex-linked. However, the closeness in dormitories, refectory settings, and poor ventilation of dormitories due to fewer vents and a large number of students, poor hygienic conditions, and other factors may enhance the spread of pathogens.

Our results showed that there was a significant difference (p<0.05) in the infection rate vis-à-vis the different age-groups. The age-group of 10-13 years was the most infected (67.9%), followed by the age-group of 14-17 years (19.6%). The age-group of 18-21 years were the least infected (12.5%) (Table 2). These findings may suggest that the younger age-groups are at a higher risk than the older age-groups. In a community-based study in the Gambia, Hill *et al*. also documented a higher prevalence of respiratory pathogens in young children than in adults (14). According to Sazawal *et al*., young children are the most vulnerable to cold (2), and therefore, these diseases usually spread rapidly through schools and households with youngsters. Common cold viruses, which are principal initiators of RTIs, are transmitted through close contact when children rub their runny noses; thus, picking up the pathogens on their fingers, which can then be transferred to others when they touch hands (33). In their study, the National Medicine Information Centre could not ascertain why common colds turn to be seasonal, but speculated that the congregation of children in schools during the colder months may be an important factor in exacerbation of RTIs (33). Cold weather characteristics of Buea may dry the lining of the nasal passages and increase susceptibility to viral invasion. It may be more serious in the young, the elderly, or in people with chronic diseases, such as diabetes, endocarditis, or chronic obstructive pulmonary diseases, or those whose immune system has been weakened by chemotherapy or disease (33). The younger age-groups in our study were more exposed to predisposing factors, such as common cold, overwork, loss of sleep, poorly-ventilated rooms, and under-nourishment, all of which lower body resistance giving way for the virus and which predispose pathogenic bacteria already present in the respiratory passages to cause infection.

The results of the antibiotic susceptibility study generally revealed that gentamicin (92%) and cefuroxime (88.4%) were the most effective antibiotics against the isolates (Table 3). *H. influenzae* showed a resistance rate of 4% to gentamicin while *K. pneumoniae* and *S. aureus* each recorded 2%. The major selective force favouring the emergence of antibio-tic resistance is their extensive use (21). It is noteworthy that gentamicin is probably less abused than other antibiotics because of its mode of administration (solely by injection) and the prohibitive cost of procurement. Cefuroxime is a relatively new antibiotic in this community and is also very expensive. These factors may be responsible for the low rate of resistance recorded for these drugs. The high rate of resistance to penicillin (100%) and co-trimoxazole (76.8%), which are commonly bought over-the-counter in drug stores, contrasts with the marked levels of susceptibilities of the isolates to gentamicin, cefuroxime, and cefazolin, which are less-frequently used, thus suggesting a relationship between antibiotic use and the level of drug resistance encountered in this study as previously suggested in another study (21).

In Omam, Kristensen and Mortensen reported a 33% resistance rate for *H. influenzae* to ampicillin (34); Gazi *et al.* documented a percentage of 20.9 in Turkey (35); and in Britain and Denmark, resistance rates of 6.2% and 5% were respectively reported for the same organism and drug (34). Compared to the 60% resistance reported in our study, we may not be able to ascertain the unusual discrepancies noted for the different regions but are led to suggest that antibiotic susceptibility patterns vary with time and geographical location as we previously documented (22). We further speculate that there may be a high level of abuse of ampicillin in the Buea area of Cameroon where the drug is cheap and readily available to the local population, thus heralding the emergence of resistant strains of organisms to the drug. In fact, all of our isolates demonstrated high levels of resistances to ampicillin. This corroborates the finding of Koulla-Shiro *et al*. who had previously documented high levels of resistance of ampicillin to their isolates of *K. pneumoniae, S. aureus,* and *S. pneumoniae* in Yaoundé, Cameroon (28).

In conclusion, the student population in boarding schools in the Buea Municipality are at risk of acquiring RTIs from their peers. They could also serve as reservoirs for the transmission of *H. influenzae* to young unvaccinated children in the community, which could lead to serious influenza and associated health consequences. This calls for an urgent need to introduce the Hib conjugate vaccine in Cameroon. Although the number of isolates used may be too small to draw meaningful conclusions on the susceptibility patterns, they, however, provide baseline data for future studies, especially considering the fact that no such data have been reported in this locality despite the very high rate of antibiotic misuse. The results of these findings are, therefore, of clinical and epidemiological significance.

## ACKNOWLEDGEMENTS

The University of Buea Postgraduate Support Scheme funded this study partly. The authors remain indebted to the schools that participated in the study.
